# Crowding-facilitated macromolecular transport in attractive micropost arrays

**DOI:** 10.1038/s41598-017-01248-8

**Published:** 2017-05-02

**Authors:** Fan-Tso Chien, Po-Keng Lin, Wei Chien, Cheng-Hsiang Hung, Ming-Hung Yu, Chia-Fu Chou, Yeng-Long Chen

**Affiliations:** 1 Institute of Physics, Academia Sinica, Taipei, Taiwan, ROC; 2 Department of Life Science, National Dong Hwa University, Hualien, Taiwan, ROC; 3 Department of Physics, National Taiwan University, Taipei, Taiwan, ROC; 4 Department of Chemical Engineering, National Tsing-Hua University, Hsinchu, Taiwan, ROC

## Abstract

Our study of DNA dynamics in weakly attractive nanofabricated post arrays revealed crowding enhances polymer transport, contrary to hindered transport in repulsive medium. The coupling of DNA diffusion and adsorption to the microposts results in more frequent cross-post hopping and increased long-term diffusivity with increased crowding density. We performed Langevin dynamics simulations and found maximum long-term diffusivity in post arrays with gap sizes comparable to the polymer radius of gyration. We found that macromolecular transport in weakly attractive post arrays is faster than in non-attractive dense medium. Furthermore, we employed hidden Markov analysis to determine the transition of macromolecular adsorption-desorption on posts and hopping between posts. The apparent free energy barriers are comparable to theoretical estimates determined from polymer conformational fluctuations.

## Introduction

Efficient transport of large particles and molecules in crowded environments is essential for biological processes in cells^1, 2^ and for molecular separation^3, 4^. Experiments that probed how the medium density and pore size distribution affect macromolecular transport in dense porous agarose gels and micropost arrays^3–5^ find hindered macromolecular transport. In an environment of repulsive obstacles, theoretical approaches have explained hindered transport by free energy barriers that slow transport when the molecular size is comparable to the obstacles^6, 7^, similar to slow transport processes in glasses and colloidal suspensions^8, 9^. A few counter-examples found crowding enhances transport under special or extreme conditions, such as rod-like molecules diffuse faster in the direction of molecular alignment in denser nematic liquids^10^. In orientationally isotropic conditions, a very recent model showed that increased crowding density could facilitate macromolecular transport in a micropost array that attracts macromolecules^11^.

In complex environments such as within a living cell, a macromolecule could interact with organelles, proteins, and the cytoskeletal network, and its transport dynamics is more complex. Macromolecular trafficking in cells plays important roles in disease development and targeted drug delivery^12–14^. Understanding intra-cellular macromolecular migration could, for example, improve the delivery efficiency of large drug molecules or suggest new designs for delivery vessels^2, 15^. This study’s goals are to verify enhanced molecular transport with more crowding and also to understand the transport of macromolecules in a crowded interactive environment by combining single-molecule tracking and computational modeling.

To emulate an interactive environment, we exploited a phenomenon in which DNA molecules (contour length 21 μm) much longer than the persistence length (≈50 nm) weakly adsorb to channel edges in nanoslits^3, 16^. In a Pyrex glass nanoslit with height *h* < 150 nm, DNA physi-sorbs to the slit edges and exhibits quasi-one dimensional diffusion^17–19^, possibly due to induced DNA polarization caused by strong static electric-field gradients at the edges. In the nanoslit micropost array device, DNA molecules appeared to weakly absorb to the posts, fluctuate in conformation and “hop” across different posts under no external fields^17, 20^. This “hopping” motion may be coupled to rare large DNA conformation fluctuations that occur in a very short time frame, although limitations in observation sampling frequencies does not allow us to resolve the process.

To systematically investigate how the crowding environment affects transport dynamics, we varied the post gap spacing in the micropost arrays. We employed single-molecule methods for tracking DNA motion in nanoslits^17, 20–22^. By controlling the crowding density and DNA-environment interactions, we sought to relate the transport probability with the energy landscape. With the ability to take images only at fixed time intervals that may be longer than needed to capture very short-lived large conformation fluctuation events, we developed a hidden Markov model to determine the hopping probability from DNA trajectories and to infer the energy landscape in this crowded environment^5, 23^.

## Results

**Figure 1:**
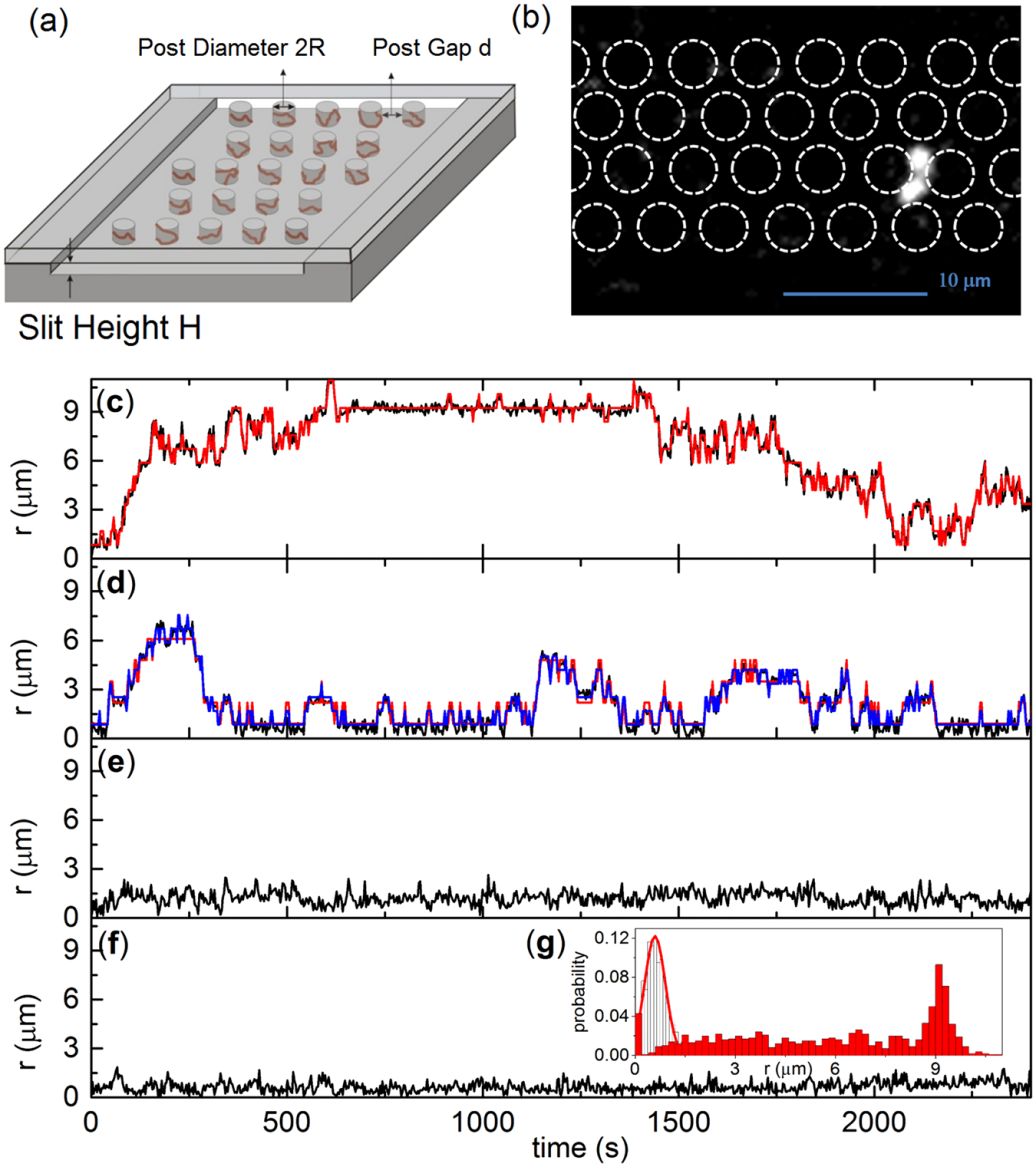
Nanoslit post array and DNA COM displacement trajectories. (**a**) Schematic of the nanoslit post array device. Red lines at post edges illustrate trapped DNA molecules. (**b**) A fluorescent DNA image in the *d* = 1.6 μm post array with the dashed circles drawn to highlight the posts. The red diamond shows the unit cell for the hexagonal array. The COM displacement trajectories are shown for *d* = 1.6 μm (**c**), 2.2 μm (**d**), 3.0 μm (**e**), and 3.6 μm (**f**). Each trajectory represents a single DNA molecule. Experiments are black Flines and HMM fits are red (with step size 0.84) and blue lines (with step size 1.3). The histogram in (**g**) shows *P*(*r*) for *d* = 3.6 μm with a bin size of 0.1 μm and 800 counts, as well as the fit with a shifted-Gaussian function (red line). The red columns show *P*(*r*) for *d* = 1.6 μm with a bin size of 0.2 μm and 1000 counts.

We fabricated nanoslit post arrays with height *h* = 65 nm, characterized in Supplementary Information S1. Previous studies found that DNA molecules adsorb to the micropost edges in nanoslits smaller than 150 nm^17, 19, 24^. The 3.5 μm diameter posts were arranged in a hexagonal array (Fig. 1a). Our preliminary studies indicated a low frequency of large displacement (“hopping”) events, and so we performed both short- and long-term experiments to capture multiple hopping events. We used devices with post gap spacing *d* = 1.6, 2.2, 3.0, and 3.6 μm for long-term (3000 s at 3 s intervals) experiments and devices with *d* = 1.0, 2.0, 3.2, and 3.6 μm for short-term (100 s at 0.5 s intervals) experiments. The YOYO-1-labelled bacteriophage λ DNA (48.5 kilobase pairs), imaged as shown in Fig. 1b, has a contour length of 21 μm and the projected radius of gyration in the nanoslit is *R*_g_ ≈ 0.84 μm^17^. DNA trajectories were obtained from the center-of-mass (COM) position $(\mathop{r}\limits^{\rightharpoonup }(t))$ and displacement $(r(t)=|\mathop{r}\limits^{\rightharpoonup }(t)-\mathop{r}\limits^{\rightharpoonup }(t=0)|)$ from the DNA images (Fig. 1)^20^. The mean squared displacement ${\rm{MSD}}({\rm{\Delta }}t)= < {[\mathop{r}\limits^{\rightharpoonup }(t+{\rm{\Delta }}t)-\mathop{r}\limits^{\rightharpoonup }(t)]}^{2} >$ was calculated over the time window Δ*t*.

Large “hops” were evident in and DNA displacement trajectories for the small gap post arrays (Fig. 1c and d). We also found the hopping probability depends on the post array gap size. The trajectory for *d* = 3.6 μm exhibited no stepwise hopping events within the observation time (Fig. 1f); the DNA molecule remained trapped near a single post as found previously^19, 24^. Visually, the adsorbed DNA has a layer thickness around 300–500 nm in the *d* = 3.6 μm array, with the radius of gyration comparable to a non-adsorbed DNA. We could not characterize the layer thickness more precisely due to fluorescent blurring and the difficulty of accurately positioning the post boundary during fluorescence imaging. The large layer thickness suggests that DNA molecules are weakly adsorbed to the post. For the adsorbed DNA, small trajectory fluctuations are mainly due to thermal fluctuations of DNA segments around a post. These fluctuations followed a shifted-Gaussian distribution with width ≈0.31 μm (Fig. 1g). For *d* = 1.6 (Fig. 1c) and 2.2 (Fig. 1d), the COM trajectories exhibited displacements that were substantially larger than that for the *d* = 3.6 μm array, resulting in much broader displacement distributions (Fig. 1f).

**Figure 2:**
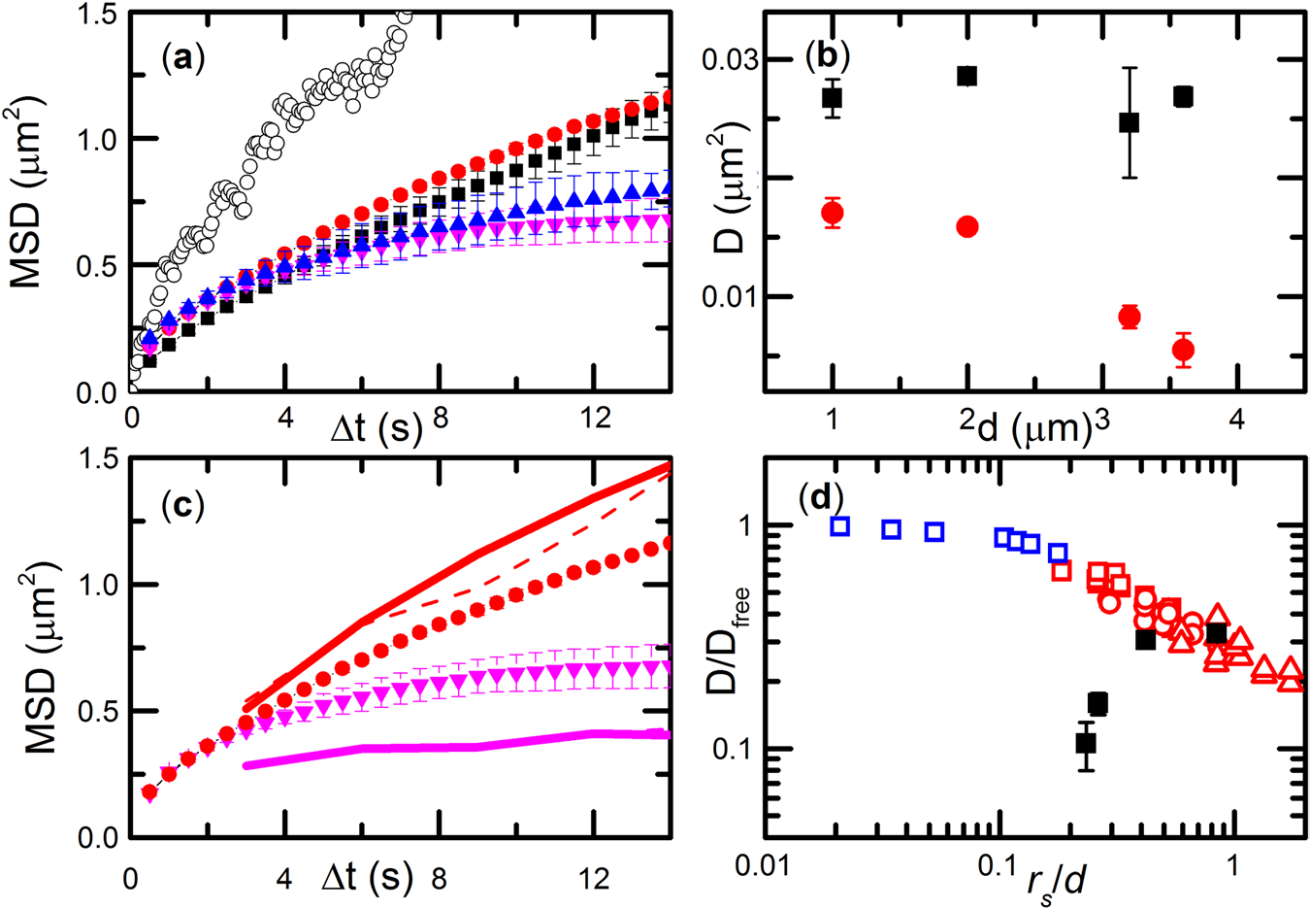
MSD and polymer diffusivity in post arrays. (**a**) Average COM MSD were obtained for *d* = 1.0 μm (black), 2.0 μm (red), 3.2 μm (blue), and 3.6 μm (magenta) post arrays. The control (open circles) was measured in the absence of the post array. COM trajectories were obtained by averaging over at least 30 short-term measurements. Error bars denote the difference between two half-ensembles. (**b**) *D*_S_ (black squares) and *D*_L_ (red circles) diffusivity in different post arrays. Error bars mark the difference between two ensemble bins. *D*_S_ is extracted from MSD (Δ*t* = 0.5 to 3 s) and *D*_L_ is extracted from MSD (Δ*t* = 3.5 to 14 s). (**c**) Comparison of the average COM MSDs obtained from short-term measurements for *d* = 2.0 (red circles) and 3.6 (magenta triangles) μm and from long-term measurements for *d* = 2.2 (red solid line) and 3.6 (magenta solid line) μm. MSD(Δ*t*) obtained from the stochastic traces for *d* = 2.2 (red dashed line) and 3.6 (magenta dashed line) μm are also depicted. (**d**) Comparison with the relative diffusivity dependence for nanoparticles in ref. 23 (blue squares), proteins in 3.9% (red squares), 5.6% (red circles), and 7.4% (red triangles) agarose gel in ref. 3, and DNA in the nanoslit post array (filled black squares). The characteristic spacing between gel fibers in agarose gels was determined from gel permeability.

The “trap-hop” motion observed in long-term DNA trajectories (Fig. 1) has interesting consequences for short-term DNA diffusion (Fig. 2). DNA MSD in the post array strongly depends on *d* and displays short- and long-term diffusive regimes (Fig. 2a), similar to colloidal diffusion in dense colloidal suspensions^25^. We determined the short- and long-term diffusivities (Supplementary Information S2). The short-term diffusivity characterizes DNA diffusion around the post perimeter and varies weakly for different values of *d* (Fig. 2b). In contrast, the long-term diffusivity characterizes hopping and increases significantly as post spacing decreases from 3.6 μm to 1.6 μm.

The normalized DNA diffusivity in Fig. 2b displayed a trend opposite of the particle diffusivities in dense agarose gels and nanopost arrays^4, 26^, in which the particle diffusivities decreased as crowding density increased. As shown in Fig. 2d, we compared DNA COM diffusion in the interactive nanoslit post array to recent studies of particle diffusion in crowded systems with purely repulsive obstacles. Prior measurements of 400 nm nanoparticle diffusion in a nanopost array (*h* = 10 μm, post diameter = 500 nm, and *d* = 1.2–4 μm) showed the relative particle diffusivity (scaled by the free diffusivity *D*_free_ without the post array) decreased as the ratio between the particle radius (*r*_s_) and *d* increased^26^. Another study of globular protein diffusion in agarose gels found more hindered protein diffusion at higher agarous concentrations, corresponding to smaller intra-gel free space^4^. These studies exhibited an opposite qualitative trend compared to current measurements, in which DNA diffusivity increased in denser post arrays due to the DNA-post attraction. Interestingly, the DNA diffusivity in the most crowded array agrees quantitatively with the particle diffusivities for the same size ratio. This suggest that the free energy barriers in the repulsive and attractive post arrays may be similar in the most crowded post array.

**Figure 3:**
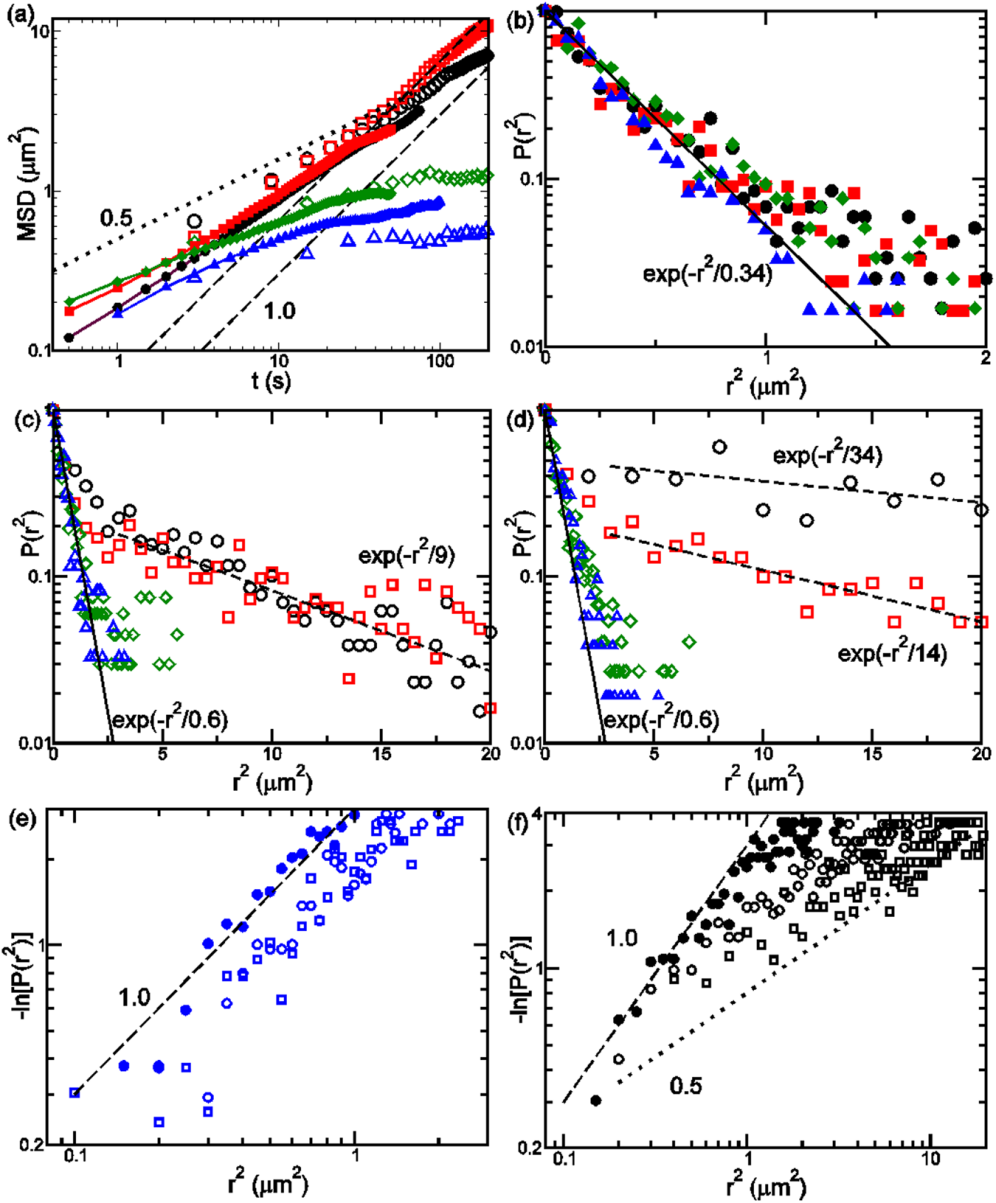
DNA MSD and COM displacement distributions for d = 1.6 (black circles) and 2.2 (red squares), 3.0 (green diamonds), and 3.6 (blue triangles) μm. (**a**) Short and long-term dynamics exhibited in the MSD. Smaller filled symbols are the short time MSD data for 1.0 (black), 2.0 (red), 3.2 (green), and 3.6 (blue) μm. The dashed and dotted lines illustrate power law exponents of 1 and 0.5, respectively. (**b**–**d**) The observed normalized distributions of *r* ^2^ for Δ*t* = 3 (**b**), 300 (**c**), and 1000 (**d**) s. In (**b**–**d**), the solid and dashed lines are drawn for fits with exp[−*r* ^2^/λ]. (**e**) and (**f**) show the log distribution functions for Δ*t* = 3 (filled circles), 30 (empty circles), and 100 s (squares) for *d* = 3.6 and 1.6 μm, respectively.

The MSD exhibits multiple power-law dependences on time shown in Fig. 3a. We can distinguish different regimes for short (t < 5 s), intermediate (5 < t < 50 s), and long (t > 50 s) time dynamics. From the short-term measurements, MSD ~ *t* ^0.5±0.1^ for short times in the *d* = 3.2 and 3.6 μm post arrays. This is qualitatively consistent with polymer internal segmental relaxation predicted in the Rouse model. In both large gap post arrays, the MSD reach plateau values for t > 30 s in both short- and long-term experiments, corresponding to DNA trapping on a single post. In comparison, in the small gap arrays of *d* = 1.0 and 2.0 μm, MSD ~ *t* ^0.7±0.1^ for short time indicating a transition from segmental relaxation to COM normal diffusion. In the intermediate regime, the MSD for both cases exhibit *t* ^0.6±0.1^ dependence, suggesting both DNA transport around a post and between posts. In the long time regime, the long-term experiments in *d* = 1.6 and 2.2 μm post arrays show that MSD ~ *t* ^0.9±0.1^ as the COM transport becomes normal diffusion. The multi-stage dynamics is qualitatively similar to non-Fickian diffusion observed for colloidal particles in nearly glassy media, where “caged relaxation” separates short and long time transport. The slow internal polymer segment relaxation at short times is observable due to strong confinement in the nanoslit and the post attraction.

For Brownian diffusion, the molecular displacement follows Gaussian distribution, i.e. *P*(*r* ^2^, Δ*t*) is proportional to exp[−*r* ^2^/*λ*], where $r{({\rm{\Delta }}t)}^{2}={[\mathop{r}\limits^{\rightharpoonup }(t+{\rm{\Delta }}t)-\mathop{r}\limits^{\rightharpoonup }(t)]}^{2}$ is the COM square displacement over a timeframe Δ*t*. For two-dimensional random walk, *λ* = 4*D*Δ*t* and *D* is the characteristic diffusivity. At short times, Fig. 3b shows that *P*(*r*(Δ*t* = 3 s)^2^) follows exponential decay with *λ* ≈ 0.34 ± 0.05. The corresponding diffusivity is 0.023 to 0.033 μm^2^/s, which is comparable to the short time diffusivity determined in Fig. 2b. In comparison, for Δ*t* = 300 and 1000 s (Fig. 3c and d, respectively), the small displacement distribution could both be fit by *λ* ≈ 0.6. This indicates that the COM displacement reached a plateau value and may be attributed to trapped DNA transport around a single post. The distribution functions for *d* = 1.6 and 2.2 μm exhibit very long tails that follow exp(−Δ*t*/*λ*_2_). These long tails are attributed to DNA transport between posts. For Δ*t* = 300 s *λ*_2_ = 9 ± 3. For Δ*t* = 1000 s, *λ*_2_ = 34 ± 5 and 14 ± 2 for *d* = 1.6 and 2.2 μm, respectively. These characteristic transport coefficients respectively correspond to *D*_2_ = 7.5 to 9.5 × 10^−3^ and 3 to 4 × 10^−3^ μm^2^/s, which are approximately half of the estimated long time diffusivity in Fig. 2b. The quantitative difference may be due to the reduced statistics for large displacements.

Taken together, these observations are consistent with the model predictions^11^ and suggest that DNA motion in dense post arrays exhibits two mechanisms characterized by *P*(*r*(Δ*t*)^2^, Δ*t*) = *C*_1_exp[−*r*(Δ*t*)^2^/*λ*_1_(Δ*t*)] + *C*_2_exp[−*r*(Δ*t*)^2^/*λ*_2_(Δ*t*)]^27^. *λ*_1_ characterizes transport around the post, *λ*_2_ characterizes transport between posts, and *C*_1_ and *C*_2_ are constants. However, an alternative interpretation is that the long-tailed distributions could be considered as stretched exponential, i.e. −ln[*P*(*r*(Δ*t*)^2^, Δ*t*)] ~ (*r*(Δ*t*)^2^)^*β*^. Figure 3e and f show that the exponent *β* remains close to 1 for *d* = 3.6 μm, which correspond to simple diffusion around the post. For *d* = 1.6 μm, *β* varies from nearly 1 to 0.58 as Δ*t* increases 3 s to 300 s, corresponding to the transition from short-term diffusion around a post to long-term combination of around-post and cross-post transport.

## Hidden Markov Analysis

From the DNA displacement analyses, we identified two dominant transport processes – DNA translation along the post perimeter and DNA translation across posts. However, photo-induced DNA cleavage from continuous imaging limits the frame rate, thus preventing direct observation of post-crossing events that could allow us to map the free energy landscape. To relate the transition mechanisms observed in the COM trajectories to post-crossing free energy barriers, we employed a hidden Markov model (HMM)-based Viterbi algorithm to model the trajectory as a series of transition events.

We considered the energy landscape to have *N* equivalent trapped steps, corresponding to the array periodicity and the limits of the finite observation time^5^. We modeled the process as a time series with trapping probability *P*_*trap*_ and transition probability *P*_*hop*_ = 1 − *P*_*trap*_, and we found the most likely transition probability P* for transport across posts. The key fitting parameter is the hopping step size *g*. Comparisons in Fig. 1 showed HMM fitted the observed trajectories very well. In post arrays with *d* = 1.6, 2.2, and 3.0 μm, the Viterbi iteration found an optimal step size for *g* = 0.6 to 1.0, which is close to *λ*_*1*_^1/2^ and also the DNA radius of gyration in the nanoslit. This indicates that the transport mechanism could be related to diffusion around a post. As shown in Table 1, the corresponding optimal trapping probability *P*_trap_* ≈ 0.55, 0.6, 0.75, and 1.0 for *d* = 1.6, 2.2, 3.0, and 3.6 μm, respectively. As expected, *P*_trap_* increased as *d* increased, with the polymer more likely to escape from a post for smaller gap sizes. For *d* = 2.2 μm, we found a second optimal fitting step size near *g* = 1.4 and *P*_trap_* ≈ 0.75, which may reflect polymer crossing posts. Although the polymers in the *d* = 1.6 μm array also move between posts, we did not find a second optimal step size, which may be due to the transport barriers too similar for us to distinguish. For the larger gapped arrays, post crossings are too rare within the observation timeframe, indicating large energy barriers.

## Free Energy Barriers of Post Crossing

The Boltzmann equation connects the transition probability *P*_hop_* to the effective free energy barrier *ΔG*, *P*_hop_^*^ ~ exp(−*β*Δ*G*), where *β* = 1/*k*_B_ *T*, *k*_B_ is the Boltzmann constant and *T* is the absolute temperature^28^. From the observed DNA COM trajectories and our HMM analysis, we estimated the effective transport free energy barriers to be *β*Δ*G* ≈ 0.8, 0.9, and 1.4 for *d* = 1.6, 2.2, and 3.0 μm, respectively. The estimated barriers are comparable to the thermal energy and increase as post spacing increases. This indicates that in addition to the enthalpic DNA segment-post adsorption, there is an entropic contribution from DNA conformational fluctuations that is required to enable a DNA molecule to reach other posts across the gaps. As a simple model of this process, we consider a one-dimensional transition path picture: The desorbed DNA in coil conformation near one post undergoes a conformation fluctuation, allowing it to “reach” another post if *d* is comparable to 2*R*_g_. Assuming the ideal coil conformation for DNA, the end-to-end distance (*R*_EE_) distribution is Gaussian. Thus, the free energy profile along the one-dimensional reaction path (denoted by *x*) consists of two half-Gaussian contributions with the centers at a distance *d* apart. The total free energy is thus given by *βF*(*x*) = (*x* ^2^/2<*x* ^2^>_0_) + [(*x* − *d*)^2^/2<*x* ^2^>_0_], where <*x* ^2^>_0_ = <*R*_EE_^2^>_0_/3 = 2*R*_g_^2^. The minima are at *x* = 0 and *x* = *d* and the barrier βF* = (*d* ^2^/8*R*_g_^2^) is at *x* = *d*/2. With *R*_g_ = 0.84 μm, βF* = 0.45, 0.85, and 1.6 for *d* = 1.6, 2.2, and 3.0 μm. These predictions are in qualitative agreement with the HMM determined barriers and further support the idea that DNA conformation fluctuation is critical for the trap-hop process.

## Verification by Langevin Dynamics

**Figure 4:**
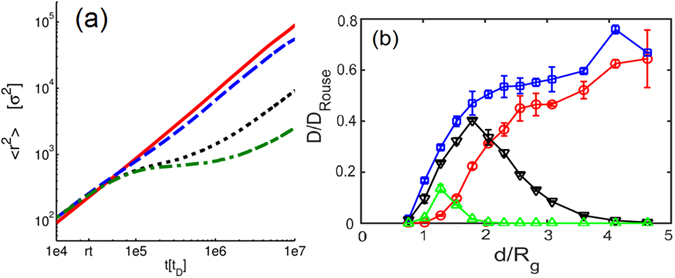
Langevin dynamics results for polymer diffusion in post arrays. (**a**) The polymer COM MSD <*r* ^2^>_xz_ in post arrays of *d/R*_*g*_ = 1.8 (solid line), 2.6 (dashed line), 3.6 (dotted-line) and 4.4 (dash-dot line). The data is calculated averaged over a time period of 10^8^ *t*_*D*_ with 10 trials for each parameter. (**b**) The dependence of model polymer diffusivity *D*_*L*_ on *d/R*_*g*_. Chain diffusivity in the post array is normalized by the bulk diffusivity. The symbols denote *ε* = 0.3 (square), 0.6 (downward triangle), 1.0 (triangle). The circles show the results in a purely repulsive array.

We tested the relationship between enhanced polymer transport and the energy landscape in the attractive post array by performing Langevin dynamics simulations^11, 29^. We chose the model parameters that best matched the experimental conditions, investigated the dependence of polymer diffusivity on the gap size *d*/*R*_g_ and the monomer-post interaction strength *ε* for an ideal polymer in a post array, and determined the MSD (Fig. 4a). In a purely repulsive array or a very weakly attractive array (*ε* = 0.3), the polymer diffusivity decreases in the more crowded array as expected (Fig. 4b). In addition, polymer diffusion is weakly affected by the gap size for *d* > 2*R*_g_ mainly due to the reduction of free space. With moderate post attraction (*ε* = 0.6 and 1.0), the model polymer moves between posts with sufficiently smaller gaps (*d*/*R*_g_ = 1.8 and 2.6 corresponding to 1.6 and 2.2 μm gaps), resulting in faster diffusion. In arrays with large gaps (*d*/*R*_g_ = 3.8 and 4.4 corresponding to 3.0 and 3.6 μm gaps), the polymer is trapped by a single post for long periods. As post-polymer attraction increases, the polymers are more strongly trapped by the posts. These qualitative trends correspond to our experimental observations.

**Figure 5:**
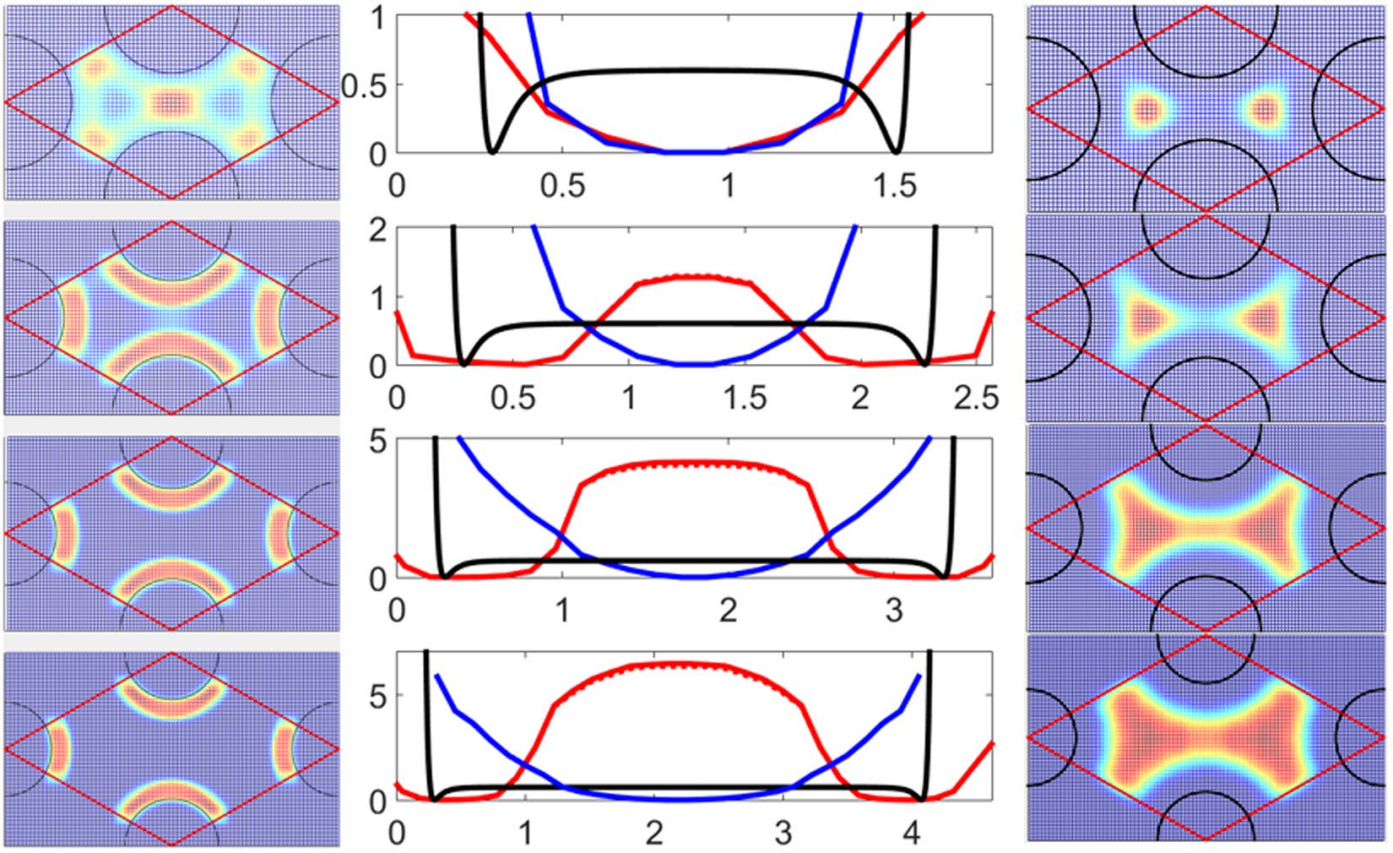
COM probability distribution and potential-of-mean-force between posts. The potential of mean force *U*_eff_ at distance *dr* from the post surface across the shortest path between two posts for *d/R*_*g*_ = 1.8, 2.6, 3.6 and 4.4 (top to bottom) for purely repulsive posts (blue line) and *ε* = 0.6 attractive posts (red line). The solid black line shows the post-bead interaction energy, and the dashed line is *U*_eff_ calculated from half of the data ensemble. The corresponding *P*(*r*_*COM*_) in a unit cell is shown for *ε* = 0.6 attractive posts (left) and repulsive posts (right). The colors blue to red indicates low to high probabilities.

In a moderately attractive post array, Fig. 4b shows the long-term diffusivity increases significantly as *d* decreases, qualitatively capturing the experimental observation. We further calculated the potential-of-mean-force *U*_eff_/*k*_B_ *T* = −ln[*P*(*r*_COM_)] using the polymer COM distribution *P*(*r*_COM_) in the post array (Fig. 5). For small gaps, *P*(*r*_COM_) peak along the post perimeter and at the center of the closest gap, suggesting the polymers are able to frequently cross between posts along these regions. In contrast, for large gaps, the post attraction slows down long term polymer diffusion drastically because the polymer cannot reach other posts. In addition, we found that the polymer COM interacts with the post over a much longer range than the post-bead attraction due to inherent intra-chain correlation, i.e. as one bead on the polymer becomes attracted to a post, the entire polymer becomes attracted to the post due to intra-polymer connectivity. The range of the COM-post potential *U*_eff_ is thus directly related to *R*_g_. As shown in Fig. 5, the post-COM attraction regions overlap for *d/R*_*g*_ = 1.8, and there is no effective energy barrier for the polymer to move between two posts. As the gap sizes increases, the energy barriers to polymer transport between posts increase significantly, as also found from the HMM analysis (Table 1). The large free energy barriers result in “trapped” DNA restricted in the near-post region. As the gap becomes smaller, the free energy barrier disappears as the DNA molecules are able to bridge across two posts by large conformation fluctuations, thus “hop” away from the initial post.

**Table 1:** *P*_trap_* values obtained from the Viterbi iteration for the best fits with *g* and *w*_*trap*_.

*d*	*P*_trap_*	*N*	χ^2^/υ	ΔG(*k*_B_ *T*)
1.6 μm	0.55	14	1	0.8
2.2 μm	0.6 (1^st^ crossing)	10	1	0.9
2.2 μm	0.75 (2^nd^ crossing)	10	1	1.4
3.0 μm	0.75	3	1	1.4

*N* is the number of trapped steps during the observation period.

In contrast, the free energy landscape for polymer transport in a repulsive array does not exhibit barriers. Thus, as the post array becomes denser, polymer transport severly slows due to the reduction in the free volume. The COM distribution becomes constrained to the center of the gap due to the polymer correlation hole near the repuslive surfaces. The comparison is particular interesting with a weakly attractive post array (*ε* = 0.3), in which the polymer COM can be very near the post surface and distributed over the gap space. Thus, polymers in the weakly attractive post array are able to access more free volume than in the repulsive post array, leading to higher diffusivity as shown in Fig. 4b. This effect may be utilitzed for macromolecular transport in very confined pores or tubes to facilitate transport.

## Discussion

To summarize, our observations of DNA motion in nanoslit micropost arrays revealed that DNA COM trajectories undergo apparent “hopping” motions due to large conformation fluctuations and segmental adsorption/desorption to the micropost array. These observations agree with the model predictions that polymer transport could be enhanced in crowded systems by controlling the polymer-environment interactions^11^. We find that there are two main transport mechanisms: DNA diffusion around an attractive post and DNA conformation fluctuation related crossing between posts. The “hopping” process is uniquely due to large conformational fluctuations accessibly with DNA molecules and large floppy macromolecules, in contrast to the adsorption/desorption of small compact macromolecules from charged patches at a liquid-solid interface^30–35^.

We also elucidated the relationship between the energy landscape and the “trap-hop” behavior with Langevin dynamics simulations and HMM analysis. We determined the most likely hopping probabilities dependent on post array density and found that the free energy barriers increased as inter-post spacing decreased. This is confirmed in Langevin dynamics simulations for polymers in an attractive post array, which also shows faster polymer diffusion in a more crowded interactive environment. In addition, the simulations also showed polymer transport is faster in the weakly attractive post array due to reduced polymer depletion region near the posts compared to non-attractive post arrays.

These findings could have important implications for controlling macromolecular mobility in crowded porous medium such as a gel, or for manipulating macromolecular transport in the cytoplasm of cells. To more realistically emulate *in vivo* crowding conditions, future studies would aim to better control the post-molecule interaction by coating nanoslit micropost arrays with biomacromolecules. This phenomenon may also be combined with other micro-channel functional elements to more effectively harvest macromolecules in novel field-free devices.

## Methods

### Nanoslit fabrication

We fabricated the nanoslit devices by first patterning Pyrex 7740 glass wafers (Corning) through standard photolithography. Briefly, wafers were spin-coated with photoresist (S1813, Shipley), exposed to ultraviolet light, and developed using MF-319 (Shipley). Inductive reactive-ion etching with CF_4_ gas was then employed to etch the glass to the desired depth. Finally, the glass was thermally fused with a 0.17-mm Pyrex 7740 coverslip (Corning) at 650 °C^17, 20^. Surface roughness of the nanoslit post array was characterized by Surface Profiler as shown in SI S1.

### DNA solution preparation

Bacteriophage λ DNA molecules (NEB) were stained with YOYO-1 (Invitrogen) at a dye:base pair ratio of 1:4. The buffer solution contains 0.5X Tris/borate/ethylenediaminetetraacetic acid (Sigma) and 10 mM NaCl (Sigma)^17, 20^. The ionic strength is ≈35 mM monovalent ions. DNA images were taken using an EMCCD camera (Ixon-897, Andor) and fluorescence microscopy (Olympus IX71 fluorescence microscope) (Fig. 1b). DNA molecules were loaded into the nanoslit by applying an electric field (300 V/cm). The field was turned off and the field-extended DNA conformation returned to equilibrium in the absence of external forces for 30 min before measurements. To reduce photobleaching and photocleaving of DNA molecules, long-term (1 frame/3 s for 3000 s) and short-term (2 frames/s for 100 s) observations were performed separately. The attraction of DNA to the edge of a nanoslit was observed previously^17, 19^. As the nanochannel surface and DNA molecules were both expected to be negatively charged in water, our observation of DNA-post attraction was surprising. We previously reported the edge-trapping of DNA molecules in a nanoslit micropost array for *H* < 150 nm^17^. In larger nanoslits, DNA molecules were not found to absorb to the edges.

### Hidden Markov model (HMM) analysis

**Figure 6:**
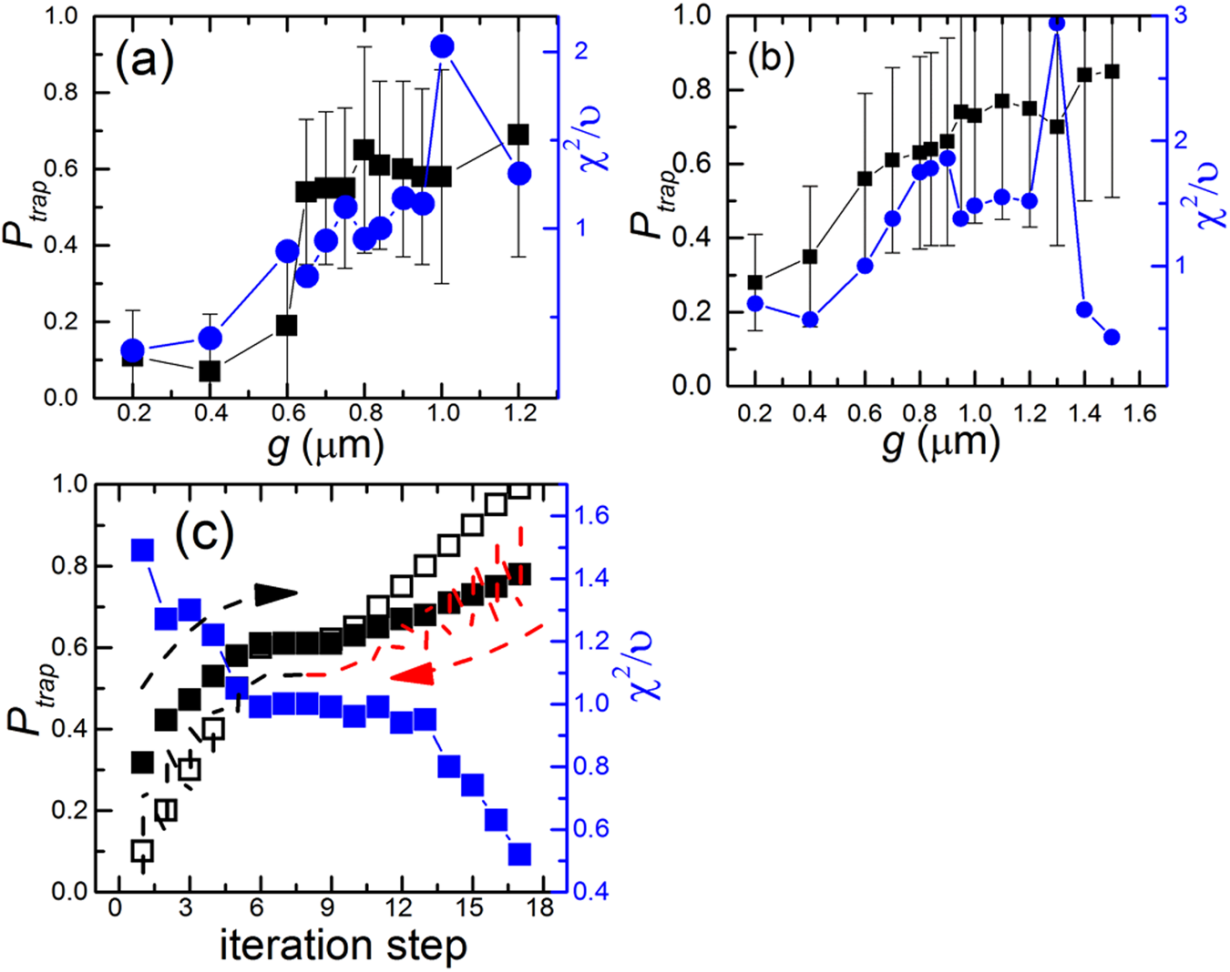
Systematic testing of optimized regimes in the Viterbi fitting at different trapped step sizes (*g*). The optimal values of *P*_*trap*_ (black) and the fit quality (χ^2^/υ, blue) were obtained by changing the trapped step size in the Viterbi fitting routine to the displacement trajectory obtained at (**a**) *d* = 1.6 μm and (**b**) 2.2 μm. Dashed lines indicate χ^2^/υ = 1, where the fitting routine yields optimized results. (**c**) The values of the input *P*_*trap*_ (open black square), the output *P*_*trap*_ (solid black square), and the goodness of the fit (solid blue square) were obtained in individual iteration steps. The black arrow with black dash lines indicates the iteration process starting from 0.1 till it reaches the converged *P*_*trap*_. The iteration starting from 0.95 are shown as red arrow with red dash lines.

The DNA images were taken at regular time intervals, which limits the observation of fast “hopping” dynamics due to DNA conformational fluctuations. The smallest detectable *P*_*trap*_ = exp(−Δ*t* * *f*_*Nyq*_), where *f*_*Nyq*_ is the Nyquist frequency = frame rate/2, or exp(−2) = 0.135^36^. In each time instance of the observed trajectories, a DNA molecule is either trapped or hopping. The thermal fluctuations of a trapped DNA are characterized by the displacement distribution function *G*_*j*_(*r*) = exp[(*r* − *r*_0_(*j*))^2^/*w*_*trap*_^2^], where *r*_0_(*j*) is the displacement of step *j* and *w*_*trap*_ = 0.31 μm is the fluctuation distribution width for *d* = 3.6 μm obtained from Fig. 1g. We considered *w*_*trap*_ to be equivalent for each step centered at *r*_0_(*j*), *j* ∈ [1, N]. The step size *g* = *r*_*0*_(*j*) − *r*_*0*_(*j* *−* *1*) ≈ 0.84 μm was found to result in the best fits (Fig. 6). Interestingly, *g* also corresponds to *R*_g_ in the nanoslit, suggesting that DNA conformation fluctuations is directly related to hopping between posts.

A custom-coded HMM-based Viterbi algorithm^36^ written in LabView found the most likely trapping probability, *P*_trap_*, by iteratively minimizing the error between the experimentally observed transition matrix T* and the HMM-predicted matrix **T**. **T**_ij_ is the hopping probability from step *i* to step *j* with the step size defined by *g*. To start, we constructed a *N* × *N* transition matrix **T** ^(0)^ from a guess of *P*_trap_^(0)^ = 1 − *P*_*hop*_^(0)^, with **T**_ij_^(0)^ = *P*_trap_^(0)^ for *i* = *j*, **T**_i,i−1_^(0)^ = **T**_i,i+1_^(0)^ = *P*_*hop*_^(0)^/2, and **T**_ij_^(0)^ = 0 for |*i* *−* *j*| > 1, i.e. **T**_0,0_^(0)^ = *P*_*trap*_^(0)^, **T**_0,1_^(0)^ = *P*_*hop*_^(0)^, **T**_N,N_^(0)^ = *P*_*trap*_^(0)^ and **T**_N−1,N_^(0)^ = *P*_*hop*_^(0)^. At each time instance *t*, an array ***v***(*t*) = [*G*_0_(*r*(*t*)), *G*_1_(*r*(*t*)), …, *G*_N_(*r*(*t*))] is determined from the observed COM trajectory and the displacement distribution function *G*_j_(*r*). The trap-step time series *s* ^(0)^(*t*) = max[***v***(*t*)·**T** ^(**0**)^], i.e. the most probable step at time *t*, is then calculated. The observed transition matrix T* ^(0)^ is constructed by counting the transition between steps in *s* ^(0)^(*t*). *P*_*trap*_^(1)^ was then obtained by minimizing |T* ^(0)^ − **T** ^(0)^|. The Viterbi iteration continued until |*P*_*trap*_^(k)^ − *P*_*trap*_^(k−1)^| is smaller than a convergence criterion. The quality of the fits to the observed transition matrix T* was evaluated with Pearson’s chi-squared test^37^.

To test whether the Viterbi iteration was essential for our analysis, each trajectory data point *r*(*t*) was assigned to the closest step *s*, generating a displacement time series *s*(*t*). From the trap/hop events in *s*(*t*), a transition matrix T* was calculated. The most optimal *P*_trap_* was obtained by minimizing |T* − **T**|, where **T**_ij_ = *P*_*trap*_ for *i* = *j* and **T**_i−1,j_ = **T**_i+1,j_ = *P*_*hop*_/2. The extent to which *P*_trap_* agreed with the observed trajectory was evaluated with Pearson’s chi-squared test (5), χ^2^/υ, where χ^2^ = ${\sum ({{\bf{T}}}_{ij}-{{\bf{T}}}_{ij}^{\ast })}^{2}/{{\bf{T}}}_{ij}^{\ast }$ and υ = degrees of freedom = number of non-zero matrix elements − 3. The reduced χ^2^ value indicates the quality of the fit to the observed transition matrix.

We determine whether the Hidden Markov model fits the observed trajectories well with the criterion *χ* ^2^/*υ* = 1. Optimal values for *P*_trap_* are reported in Table 1. Figure 6a and b show that the best fitting step size is *g* ≈ 0.8 μm after the Viterbi iterations. For *d* = 2.2 μm, Fig. 6b shows a second optimal step size for *g* ≈ 1.4 μm, possibly attributed to the larger separation of time scales between post-crossing and around-post transport. Without using the Viterbi iteration, *P*_trap_* values thus calculated yielded *χ* ^2^/*υ* > 5 (data not shown), indicating a poor fit to the observed trajectory. Consistency of the Viterbi iteration is also verified by checking the same *P*_trap_* is obtained whether we perform the iteration from low to high *P*_trap_ or from high to low *P*_trap_, as shown in Fig. 6c.

### Langevin dynamics simulations

The positions of all beads {***r***_i_(*t*)} on a coarse-grained bead-spring model DNA are propagated with the velocity-Verlet algorithm^11, 29^. The total force acting on each bead is ***f***_i_ = ***f***_i_^int^ + ***f***_i_^R^ + ***f***_i_^fric^, where ***f***_i_^int^ = −∇*U*_i_^int^ results from bonded and non-bonded bead-bead potential *U* ^int^(***r***), as given as

1 $$\frac{{{U}_{i}}^{int}(r)}{{k}_{B}T}=\sum _{i}-\frac{{k}_{s}{{r}_{0}}^{2}}{2}{\rm{l}}{\rm{n}}[1-\frac{|{{\boldsymbol{r}}}_{i}-{{\boldsymbol{r}}}_{i+1}|}{{{\boldsymbol{r}}}_{0}}]+4[{(\frac{\sigma }{|{{\boldsymbol{r}}}_{i}-{{\boldsymbol{r}}}_{i+1}|})}^{12}-{(\frac{\sigma }{|{{\boldsymbol{r}}}_{i}-{{\boldsymbol{r}}}_{i+1}|})}^{6}]$$

The *i*_th_ bead is bonded to the (i + 1)th bead with the finite extensible nonlinear elastic (FENE) potential with spring constant *k*_*s*_ = *30* *k*_*B*_ *T*/*σ* ^*2*^ and maximum bond length *r*_*o*_ = 1.5*σ*, where *k*_*B*_ *T* = 1 is the thermal energy and *σ* = 1 is the bead diameter. Non-bonded beads do not interact and the polymer is a Gaussian chain. The friction force is ***f***_i_^fric^ = −*m*_i_ *γ* ***v***_i_, with the friction coefficient *γ* = *0*.*1* and bead mass *m*_*i*_ = 1. The fluctuation force is ***f***_i_^R^ with zero mean and variance (*2* *m*_*i*_ *γk*_*B*_ *T/*Δ*t*)^*1/2*^. A short-ranged attraction between polymer beads and the posts is modeled with

2 $$\frac{{U}_{PA}(r)}{{k}_{B}T}=4\varepsilon [{(\frac{\sigma }{|r-R|})}^{12}-{(\frac{\sigma }{|r-R|})}^{6}],r < R+{r}_{cutoff}$$

where *R* is the post radius, and *ɛ* is the post-bead interaction energy. For attractive posts, the interaction potential is set to zero for (*r* − *R*) > *r*_cutoff_ = 2.5σ, providing an attractive energy minimum of depth *εk*_B_ *T*. For repulsive posts, the attraction is removed by choosing *r*_cutoff_ = 1.12*σ*. The post array is bounded by walls with the height *H* = 3*σ*. The reflective boundary condition is used.

The bead diffusivity *D*_*0*_ = *k*_*B*_ *T/m*_*i*_ *γ*. The physical characteristic time is the diffusion time of a bead over one bead diameter *t*_*D*_ = *σ* ^*2*^ */D*_*0*_ = *σ* ^*2*^ */*(*k*_*B*_ *T/m*_*i*_ *γ*) = 0.1. To ensure accurate integration, we chose Δ*t* = 0.1 *t*_*D*_. Typical simulation trials were performed for 10^7^ *t*_*D*_ to calculate the ensemble average properties. The equilibrium projected radius of gyration for a polymer with *N* = 160 beads is *R*_*g*_ = 3.9*σ*. In this free-jointed polymer model, we can match the simulation unit length *σ* to the Kuhn segment length of a YOYO-labeled DNA of ≈134 nm. Thus, the contour length of the model DNA is ≈21.3 μm and the projected *R*_g_ is ≈522 nm, which are close to the reported values for λ-DNA^1^. The equilibrium polymer diffusivity is *D*_*Rouse*_ = 0.00625[*σ* ^*2*^ */t*_*D*_] = *D*_*0*_ */N* in the free environment, as predicted by the Rouse model^7^. However, due to finite size effects in the simulation model, we chose a model slit height *H* = 3 *σ*, which is much larger than the experimental system. Our goal is only to test whether we are able capture the qualitative behavior of increased diffusivity with reduced gap size, rather than make quantitative comparisons.

To better understand enhanced diffusion in dense attractive post arrays, we performed comparisons with the experimental measurements given the same non-dimensional inter-post gap size *d/R*_*g*_ and post radius *R*/*R*_*g*_, as shown in Table 2.

**Table 2:** Model gap sizes and post diameters.

Model		Experiment	
*R* = *16 σ*	*R*/*R*_g_ = 4.1	*R* = 3.5 *μm*	*R*/*R*_g_ = 4.2
*d* = 7 *σ*	*d/R*_*g*_ = 1.8	*d* = 1.6 *μm*	*d/R*_*g*_ = 1.9
d = 10 *σ*	*d/R*_*g*_ = 2.6	*d* = 2.2 *μm*	*d/R*_*g*_ = 2.6
d = 14 *σ*	*d/R*_*g*_ = 3.6	*d* = 3.0 *μm*	*d/R*_*g*_ = 3.6
d = 17 *σ*	*d/R*_*g*_ = 4.4	*d* = 3.6 *μm*	*d/R*_*g*_ = 4.3

In Fig. 4a, the polymer COM MSD in the post array for *ε* = 0.6, *d*/*R*_*g*_ = 1.8, 2.6, 3.6, and 4.4 show several time-dependent regimes. The MSD is calculated only in the slit-plane, as is measured in the experiments. For *d/R*_*g*_ = 1.8 and 2.6, we observe the MSD is linearly proportional to time, as expected for Brownian diffusion. In contrast, the polymer MSD is linear at short and long times for *d/R*_*g*_ = 3.6 and 4.4, but it undergoes a plateau transition at intermediate times. At short times, the polymer diffuses around a single post in a Brownian manner. However, the MSD exhibit plateaus at intermediate time intervals, indicate the polymer is trapped on a post and unable to freely move between posts. The trapping interval may be extremely long because only large polymer conformation fluctuations will allow the polymer to ‘hop’ to another post. For sufficiently long times such that the polymer escapes the post trap, the MSD returns to linearly dependent on time, characterizing Brownian diffusion once again. Correspondingly, the short term chain COM diffusivity are nearly the same for all *d*, and the intermediate term chain COM diffusivity decreases as *d/R*_*g*_ increases from 1.8 to 4.4. These results agrees very well with the experimental observations of short and long term diffusivities in Fig. 3.

Although the post-bead attraction is short-ranged, the range of influence on the polymer is extended due to bead-bead connectivity. The effective attraction on the polymer can be determined from the COM distribution in the post array *P*(***r***_COM_) and the potential of mean force *U*_eff_ = −*k*_B_ *T* ln[*P*(***r***_COM_)]. The distribution function is calculated from an ensemble collected every 100*t*_*D*_ over 10^7^ *t*_*D*_ for 10 simulation trials.

## Electronic supplementary material

Supplemenatry Information
