# Supplementary material for: Crowding-facilitated macromolecular transport in attractive micropost arrays
Source: Sci Rep. 2017 May 2;7:1340. doi: 10.1038/s41598-017-01248-8 (PMC5430964; doi:10.1038/s41598-017-01248-8)
Supplement: Supplementary file 1 — Supplemenatry Information [file 41598_2017_1248_MOESM1_ESM.pdf]

Crowding-facilitated macromolecular transport in attractive micropost arrays

Authors: Fan-Tso Chien[1,2], Po-Keng Lin[1], Wei Chien[1,3], Cheng-Hsiang Hung[3], Ming-Hung Yu[1], Chia-Fu Chou[1], Yeng-Long Chen[1,3,4]*

Supplementary Information

S1. Nanoslit post array roughness characterization

We profiled the surfaces of the nanoslit devices and found the average roughness to be approximately 1 nm, as measured by atomic force microscopy (Veeco Bioscope, Bruker). Fig. S1a provides an illustration and surface profile of a nanoslit micropost array. Given the small amount of roughness, steric trapping seems unlikely to underlie the observed DNA-post attraction. Trapping events were observed only in Pyrex-glass nanochannels and not observed with silica glass (data not shown), which indicates that the trap-hop process could be driven by non-homogeneous surface-charge distributions. Sharp edges in the nanochannels may establish a static electric-field gradient, which could polarize DNA molecules and lead to dielectrophoretic attraction.[19,24]

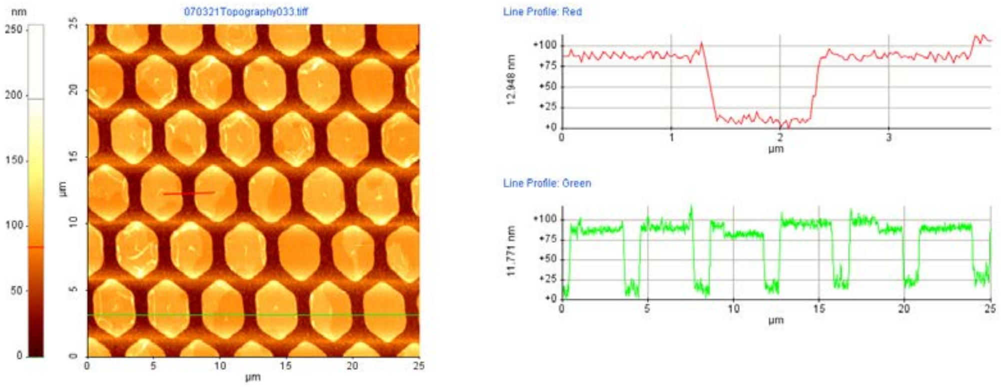

Figure S1 A nanoslit post array (post diameter 3.5 μm) with gap size d = 1.0 μm and h = 65 nm is profiled with a surface profiler. The depth profiles across the red and green lines in the left figure are shown in the right two figures with corresponding line color.

S2. Short-term COM measurements and comparisons to particle diffusivity measured in three-dimensional crowded systems

We extracted the diffusivity of DNA molecules from the MSD as a function of time for DNA in various post arrays. For normal diffusion in two dimensions, MSD = 4 D Δt. Conditions for the short-term MSD-Δt experiments are listed in Table S1. DNA COM MSD- Δt data exhibited two linear regimes (Fig. S2). We observed faster diffusion for Δt = 0-3 s, corresponding to DNA dynamics away from the microposts. Our observation that short-term diffusivity does not strongly depend on d supports this interpretation (Table S1). For Δt = 3.5-14 s, we observed slower diffusion corresponding to DNA trapping and hopping due to interactions with the posts. This long-term diffusivity strongly depends on d (Table S1).

d	Short-term diffusivity ($\mu m^2/s$)	Long-term diffusivity ($\mu m^2/s$)	Number of molecules
1.0 μm	0.0267 ± 0.0016	0.0171 ± 0.0013	54
2.0 μm	0.0286 ± 0.0004	0.0159 ± 0.001	62
3.2 μm	0.0246 ± 0.0046	0.0083 ± 0.0009	33
3.6 μm	0.0269 ± 0.0008	0.0055 ± 0.0014	52

Table S1. DNA diffusivities in different post arrays and the number of samples for MSD-Δt measurements. Means and standard deviations are reported.

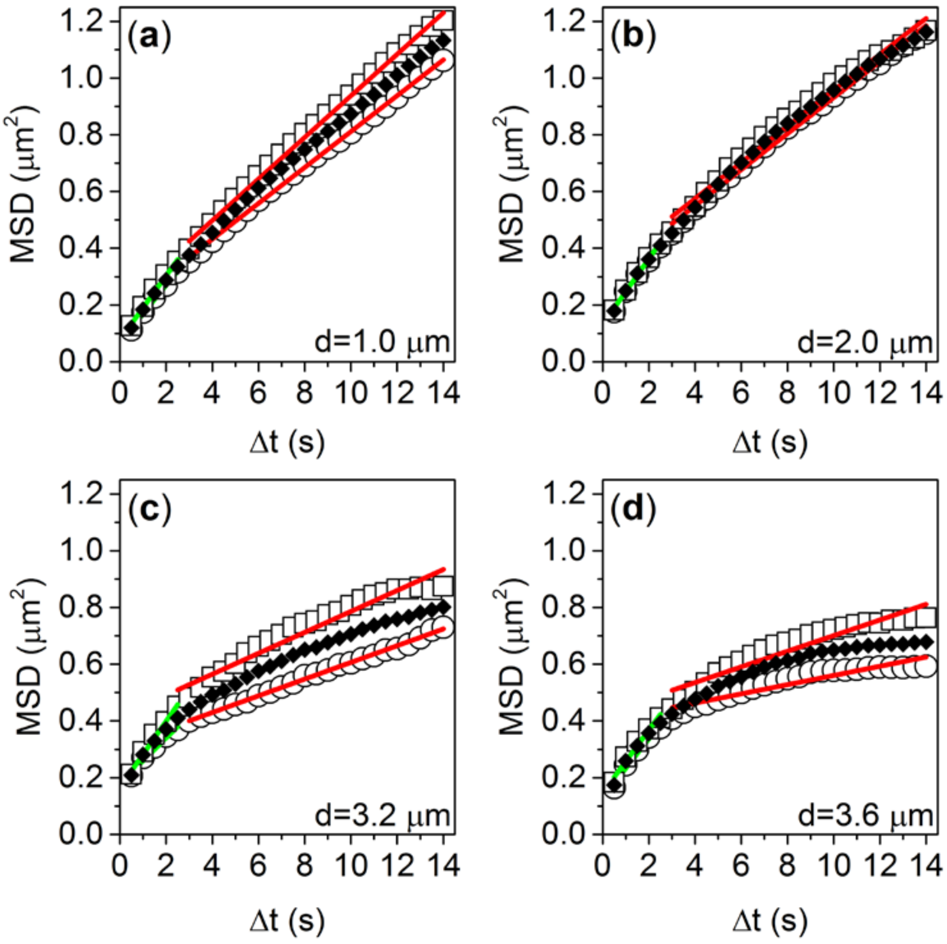

Figure S2. Diffusivity in two-stage, linear regimes captures MSD trajectories from short-term measurements at *d* = 1.0 µm (a), 2.0 µm (b), 3.2 µm (c), and 3.6 µm (d). Two equal-sized ensembles were used to calculate the MSD trajectories (black squares and black circles) and estimate the error of the MSD measurements. Linear fits to the regime between 0 s and 3 s and to the regime between 3.5 s and 14 s are shown as green lines and red lines, respectively. Filled diamonds indicate the average value of the two MSD trajectories.
